# Regulation of AMPA receptor trafficking by secreted protein factors

**DOI:** 10.3389/fncel.2023.1271169

**Published:** 2023-11-27

**Authors:** Bethany J. Rennich, Eric S. Luth, Samantha Moores, Peter Juo

**Affiliations:** ^1^Department of Developmental, Molecular and Chemical Biology, Tufts University School of Medicine, Boston, MA, United States; ^2^Graduate Program in Cell, Molecular and Developmental Biology, Tufts University School of Medicine, Boston, MA, United States; ^3^Department of Biology, Simmons University, Boston, MA, United States; ^4^Graduate Program in Cell, Molecular Biology, Developmental Biology, Graduate School of Biomedical Sciences, Tufts University School of Medicine, Boston, MA, United States

**Keywords:** AMPAR, trafficking, secreted factors, regulation, synapse

## Abstract

AMPA receptors (AMPARs) mediate the majority of fast excitatory transmission in the brain. Regulation of AMPAR levels at synapses controls synaptic strength and underlies information storage and processing. Many proteins interact with the intracellular domain of AMPARs to regulate their trafficking and synaptic clustering. However, a growing number of extracellular factors important for glutamatergic synapse development, maturation and function have emerged that can also regulate synaptic AMPAR levels. This mini-review highlights extracellular protein factors that regulate AMPAR trafficking to control synapse development and plasticity. Some of these factors regulate AMPAR clustering and mobility by interacting with the extracellular N-terminal domain of AMPARs whereas others regulate AMPAR trafficking indirectly via their respective signaling receptors. While several of these factors are secreted from neurons, others are released from non-neuronal cells such as glia and muscle. Although it is apparent that secreted factors can act locally on neurons near their sites of release to coordinate individual synapses, it is less clear if they can diffuse over longer ranges to coordinate related synapses within a circuit or region of the brain. Given that there are hundreds of factors that can be secreted from neuronal and non-neuronal cells, it will not be surprising if more extracellular factors that modulate AMPARs and glutamatergic synapses are discovered. Many open questions remain including where and when the factors are expressed, what regulates their secretion from different cell types, what controls their diffusion, stability, and range of action, and how their cognate receptors influence intracellular signaling to control AMPAR trafficking.

## Introduction

Forming and maintaining functional glutamatergic synapses is crucial for proper brain function as glutamatergic transmission underlies vital processes such as cognition, motor coordination, learning and memory. Dysfunction in glutamatergic synapse formation, maintenance and function has been implicated in conditions ranging from autism spectrum disorders to epilepsy to neurodegenerative disorders, highlighting the importance of glutamate signaling for brain function.

AMPA receptors (AMPARs) are the principal ionotropic glutamate receptors that mediate fast, excitatory synaptic transmission. AMPARs are tetrameric cation channels that are assembled from a combination of 4 different subunits, GluA1-GluA4 (Hansen et al., 2021). Subunit composition and association with intracellular proteins and auxiliary subunits in the membrane, like Transmembrane Associated Receptor Proteins (TARPs), confers distinct trafficking characteristics. Both composition and expression levels vary across brain regions, adding another layer of complexity. Synaptic AMPAR abundance is a major determinant of synaptic strength and is tightly regulated via multiple mechanisms including receptor synthesis, trafficking, membrane insertion, clustering, internalization, recycling, and degradation, as well as modulation of channel function (Anggono and Huganir, 2012). Many forms of synaptic plasticity engage one or more of these mechanisms to precisely control the number and function of AMPARs at the synapse.

**FIGURE 1:**
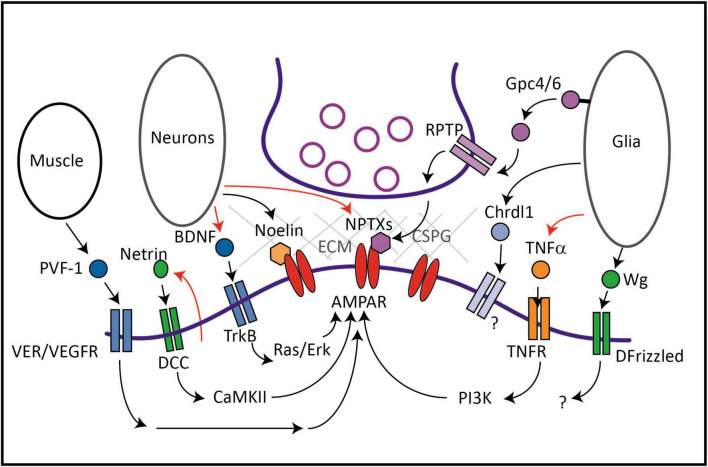
Protein factors secreted from neurons, glia or muscle regulate AMPAR trafficking and synaptic levels. Netrin is released in an activity-dependent manner from neurons and results in an autocrine activation of its receptor DCC. Downstream activation of CaMKII leads to increased delivery of GluA1-containing AMPARs to synapses. BDNF is also secreted from neurons in an activity-dependent manner and activates the tyrosine kinase receptor TrkB and downstream Ras and Erk signaling to increase AMPAR transcription, translation and synaptic incorporation. BDNF can also be secreted from glia (not shown). Noelin and NPTX are secreted from neurons, and act as extracellular scaffolds by interacting with the N-terminal domain of AMPARs and a network of proteins including ECM components. Glia such as astrocytes secrete Gpc4/6 which acts on presynaptic RPTPs leading to the release of NPTX. Chrdl1 acts via an unidentified receptor and signaling pathway to promote synapse maturation during development by inducing the switch to calcium-impermeable GluA2-containing AMPARs. TNFα is secreted from glia in an activity-dependent manner and activates the TNFR and downstream PI3 kinase to upregulate synaptic AMPARs during homeostatic synaptic scaling. In *Drosophila*, Wg is released from specialized glia that regulate glutamate receptor cluster size and synaptic localization via its receptor DFrizzled. *In C. elegans*, PVF-1/VEGF secreted by muscle promotes surface levels of AMPARs in distal neurons via its receptor VER-1 and VER-4. PVF-1 and the VERs are thought to promote surface levels of AMPARs via long-distance recycling via retromer. In mammals, VEGF also promotes AMPAR levels at synapses and can be secreted from many cells including neurons, glia and muscle, although it is not known if muscle-derived VEGF regulates AMPARs in mammals. Red arrows represent activity-dependent regulation of the secreted factor.

Much progress has been made in identifying proteins that interact with the intracellular domain of AMPARs and the mechanisms by which they regulate clustering and trafficking (Diering and Huganir, 2018). However, these processes can also be regulated by extracellular, secreted factors that impact synaptic strength and plasticity (Allen and Eroglu, 2017; Yuzaki, 2018). One class of secreted factors directly binds to the extracellular N-terminus of AMPARs and clusters receptors at synapses (i.e., Neuronal Pentraxins, Noelins), whereas another class are diffusible ligands that control AMPAR trafficking indirectly via their cognate signaling receptors (i.e., Netrin-1, BDNF, VEGF, TNFα) (Figure 1). Some of these diffusible factors are secreted from neurons, whereas others are secreted from non-neuronal cells such as glia and, intriguingly, from distal tissues such as muscle. In this mini-review, we discuss what is known about secreted proteins that regulate AMPAR trafficking and highlight some open questions that remain to be addressed.

## Factors secreted from neurons

### Neuronal pentraxins (NPTXs)

The neuronal pentraxin family consists of two secreted glycoproteins, NPTX1/NP1 and NPTX2/NP2/NARP, and the NPTX receptor (NPTXR). NPTX1 and NPTX2 cluster AMPARs at excitatory synapses onto inhibitory neurons, interacting directly with the N-terminus of AMPARs or indirectly via NPTXR (Table 1). Secreted NPTX1 interacts with NPTXR and GluA4 at shaft synapses resulting in the clustering of NPTX1 and GluA4-containing AMPARs, which are highly expressed on parvalbumin-positive (PV +) interneurons in the forebrain (Sia et al., 2007; Pelkey et al., 2015). The selective effect of NPTXs on these interneurons can be attributed to their almost exclusive interaction with GluA4 subunits (Sia et al., 2007; Pelkey et al., 2015). NPTX2 is an immediate early gene whose expression is correlated with neuronal activity (O’Brien et al., 1999; Xu et al., 2003; Chang et al., 2010) and mediates homeostatic synaptic scaling (Chang et al., 2010). NPTX2 is released from dense-core vesicles (DCVs) near glutamatergic synapses, multimerizes, and becomes trapped in the Extra-Cellular Matrix (ECM)-containing perineuronal nets that surround PV + inhibitory neuron cell bodies (O’Brien et al., 1999; Reti et al., 2008; Chang et al., 2010). Thus, NPTXs act as extracellular scaffolds that interact with and cluster AMPARs and mediate an activity-dependent mechanism to recruit inhibitory neurons into circuits to dampen excitation. Interestingly, NPTX2 can be detected in the circulatory system (Reti et al., 2008), suggesting it may have other functions and act far beyond its sites of release.

**TABLE 1:** List of secreted factors affecting AMPARs.

Secreted factor	Cell of origin	Effect on AMPARs	References
Neuronal Pentraxins	Neurons	Interacts with the NTD of AMPARs and clusters receptors	O’Brien et al., 1999; Sia et al., 2007
Netrin-1	Dendrites	Activity-dependent secretion of Netrin-1 promotes GluA1 at synapses, promotes maturation of synapses and LTP	Glasgow et al., 2018
Noelins/Olfactomedins	Neurons	Acts as an extracellular scaffold that binds NTD of AMPARs and a network of extracellular proteins, anchors and stabilizes receptors limiting their mobility, and contributes to LTP generation	Pandya et al., 2018; Boudkkazi et al., 2023
Insulin	Neurons, pancreas	Promotes AMPAR endocytosis	Lin et al., 2000; Man et al., 2000
SPARC	Astrocytes, microglia	Inhibits synaptic recruitment of GluA1 and GluA2, inhibits synapse formation	Jones et al., 2011
Thrombospondin	Astrocytes	Promotes lateral diffusion and endocytosis of AMPARs	Hennekinne et al., 2013
Glypicans	Astrocytes	Increases synaptic levels of GluA1, promotes synapse formation	Allen et al., 2012; Farhy-Tselnicker et al., 2017
Chordin-like-1	Astrocytes	Increases synaptic calcium-impermeable GluA2, limits plasticity	Blanco-Suarez et al., 2018
CSPGs	Astrocytes	Regulates AMPAR surface mobility and synaptic strength	Frischknecht et al., 2009; Pyka et al., 2011
Leucine-Rich Glioma-Inactivated Protein 1 (Lgi1)	Neurons	Promotes AMPAR levels and function in the hippocampus	Fukata et al., 2006, 2010; Ohkawa et al., 2013
Wg/Wnt, Wnt 7a	Neurons, glia	Regulates synapse development and GluR distribution at the fly NMJ	Packard et al., 2002; Mathew et al., 2005; Miech et al., 2008; Ciani et al., 2011
TNFα	Astrocytes, microglia, neurons?	Regulates AMPAR levels and synaptic scaling	Stellwagen et al., 2005; Stellwagen and Malenka, 2006; Lewitus et al., 2014
BDNF	Neurons, glia and muscle	Promotes excitatory synapse formation, increases AMPAR expression and trafficking, important for LTP; exercise increases BDNF secretion in muscle and neurons	Castrén et al., 1993; Dragunow et al., 1993; Kang and Schuman, 1995a; Messaoudi et al., 1998; Balkowiec and Katz, 2000; Jourdi et al., 2003; Aicardi et al., 2004; Caldeira et al., 2007; Cuppini et al., 2007; Matthews et al., 2009; Wrann et al., 2013; Szuhany et al., 2015
VEGF	Endothelial cells, neurons, glia and muscle	Regulates AMPAR surface levels, LTP and learning and memory	Licht et al., 2011; De Rossi et al., 2016; Luth et al., 2021
FNDC5/Irisin	Muscle and neurons	Exercise increases Irisin which increases BDNF in the brain	Boström et al., 2012; Wrann et al., 2013; Islam et al., 2021
IGF-1	Muscle and neurons	Exercise increases IGF-1 which increases BDNF	Ding et al., 2006
TGFβ/BMP	Neurons	Regulates synaptic AMPAR levels by controlling subunit expression in mammals and *C. elegans*	Bae et al., 2011; McGehee et al., 2015

### Netrin-1

Netrin-1 was discovered as a regulator of neurodevelopmental processes including cell migration, axon guidance, and synapse formation in invertebrates and vertebrates (Ishii et al., 1992; Kennedy et al., 1994; Colón-Ramos et al., 2007). More recently, Netrin-1 was shown to promote excitatory synapse formation (Goldman et al., 2013) and activity-dependent insertion of AMPARs into synapses (Glasgow et al., 2018). NMDAR-mediated Ca^2+^ influx stimulates the release of Netrin-1 from hippocampal dendrites, resulting in autocrine activation of the Netrin receptor Deleted in Colorectal Cancer (DCC), activation of Calcium/Calmodulin Kinase II (CaMKII) and increased surface abundance of GluA1-containing AMPARs (Glasgow et al., 2018, 2020). Netrin-1 released from dopaminergic and GABAergic neurons in the ventral tegmental area similarly acts in an autocrine manner to promote AMPAR-mediated currents (Cline et al., 2023). The Netrin co-receptor Down Syndrome Cell Adhesion Molecule (DSCAM) also promotes AMPAR levels and clustering in cultured *Aplysia* neurons (Li et al., 2009). Additionally, long-term potentiation (LTP) occludes Netrin-1-induced potentiation of AMPAR EPSCs, and vice versa, indicating that activity-dependent secretion of Netrin-1 is a mechanism of LTP (Glasgow et al., 2018). Conditional deletion of Netrin or DCC from excitatory neurons in the hippocampus results in defects in spatial memory in rodents (Horn et al., 2013; Wong et al., 2019), and shRNA-mediated knockdown of Netrin-1 from spinal dorsal horn neurons impairs pain sensation and decreases the membrane fraction of GluA1-containing AMPARs (Cui et al., 2021). Thus, Netrin is secreted in an activity-dependent manner from dendrites of many cell types and promotes synaptic AMPAR retention, providing an example of a secreted factor being used in an autocrine fashion to strengthen glutamatergic signaling in response to postsynaptic activity. It remains to be seen whether the effects of Netrin-1 are restricted to stimulated synapses or if secreted Netrin-1 can diffuse further away to reach neighboring unstimulated synapses.

### Noelin

Noelin-1/Olfactomedin-1 is a glycoprotein secreted from neurons in the cortex, cerebellum and hippocampus. Noelin-1 and the related Noelin-2 regulate AMPAR trafficking and function in zebrafish and rodents (Sultana et al., 2014; Nakaya et al., 2017). Noelins are conserved in all vertebrates but do not appear to have an obvious homolog in invertebrates (Boudkkazi et al., 2023). Noelin-1 was originally isolated as an AMPAR-interacting protein from rodent synaptosomes (von Engelhardt et al., 2010; Schwenk et al., 2012). Noelin-1 interacts with GluA1 and GluA2 in heterologous cells and, similar to NPTXs, binds directly to the N-terminal domain of GluA2 (Pandya et al., 2018), resulting in the stabilization of synaptic AMPARs (Díaz-Alonso et al., 2017). Interestingly, the effects of Noelin-1 are modified by the presence of the ECM because Noelin-1 reduces AMPAR mobility on young neurons but not on mature neurons which are surrounded by ECM. Noelin-1 may also link AMPARs with Chondroitin sulfate proteoglycans (CSPGs) in the ECM via Nogo, a Noelin-1 receptor (Frischknecht et al., 2009). A recent study shows that Noelins 1-3 act as polyvalent extracellular scaffolds that stabilize AMPARs by anchoring them to a network of secreted and transmembrane proteins (Boudkkazi et al., 2023). Using high-resolution immuno-EM and electrophysiology recordings, neurons lacking Noelins 1-3 were observed to have 40 and 70% reductions in AMPARs at synapses on excitatory and inhibitory neurons, respectively, and defects in hippocampal LTP. This study suggests that secreted factors like Noelins and their extracellular protein networks are major contributors to LTP (Boudkkazi et al., 2023). Interestingly, chemical LTP increases Noelin-1 levels in the synapse suggesting that activity may increase Noelin-1 secretion to stabilize synaptic AMPARs during plasticity (Pandya et al., 2018).

### Brain-derived neurotrophic factor (BDNF)

Brain-derived neurotrophic factor, a member of the neurotrophin family, signals through its tyrosine-kinase receptor, tropomyosin-related Kinase B (TrkB) to regulate several processes including neurogenesis, neuronal survival, axon outgrowth, and synapse formation (Messaoudi et al., 1998; Lee and Hempstead, 2018). LTP stimulates BDNF transcription via the transcription factor CREB (Castrén et al., 1993; Dragunow et al., 1993; Tao et al., 1998, 2002; Hong et al., 2008) and promotes BDNF secretion (Balkowiec and Katz, 2000; Aicardi et al., 2004). Conversely, application of mature BDNF induces LTP at hippocampal synapses (Kang and Schuman, 1995a,b). These early studies suggested that BDNF could regulate AMPAR expression or trafficking during LTP. Indeed, BDNF/TrkB signaling increases transcription and protein expression of GluA1, GluA2, and GluA3, and AMPAR colocalization with PSD-95 in dendrites (Jourdi et al., 2003; Caldeira et al., 2007; Nakata and Nakamura, 2007). Of all secreted factors, the intracellular signaling pathways that mediate the effects of BDNF on synaptic AMPAR levels are probably the best understood. Specifically, BDNF/TrkB complexes activate the Ras/ERK pathway that is involved in surface delivery of AMPARs (Li and Keifer, 2008, 2009; Li and Wolf, 2011; Reimers et al., 2014) and phosphorylates S831 on GluA1 subunits, a critical site for CaMKII and protein kinase C (PKC) binding, promoting AMPAR synaptic delivery and retention (Caldeira et al., 2007; Keifer, 2022). Thus, BDNF is a secreted factor whose expression and secretion are regulated by activity that promotes AMPAR levels at synapses via multiple intracellular mechanisms.

## Factors secreted from glia

### Thrombospondin-1

The large glycoprotein Thrombospondin-1 is secreted by astrocytes and was originally shown to induce synapse formation during development, although these synapses initially lack AMPARs and are silent (Christopherson et al., 2005). Thrombospondin-1 also functions in the mature nervous system to reduce synaptic transmission: treatment of cultured rat spinal cord neurons with Thrombospondin-1 increases lateral diffusion and endocytosis of AMPARs and increases levels of inhibitory glycine receptors at synapses (Hennekinne et al., 2013). Thus, Thrombospondin-1 is a glia-secreted factor that regulates synapse development and AMPAR trafficking in mature neurons.

### Secreted protein acidic and rich in cysteines (SPARC)

Astrocytes and microglia secrete the protein SPARC which antagonizes synapse formation induced by hevin, another astrocyte-secreted factor (Jones et al., 2011; Kucukdereli et al., 2011). Consistent with a role for SPARC in negatively regulating excitatory transmission, hippocampal neurons from SPARC knock-out mice exhibit increased mESPC amplitude and frequency. Moreover, when cultured with SPARC knock-out astrocytes, hippocampal neurons exhibit increased surface levels of GluA1 and GluA2. Intriguingly, secretion of SPARC from astrocytes is dependent on synaptic activity, suggesting a model whereby increased synaptic activity leads to SPARC release and downregulation of AMPAR surface levels and signaling (Jones et al., 2011). These studies indicate that SPARC is an activity-regulated secreted factor that inhibits glutamatergic signaling by reducing surface levels of AMPARs at synapses, although how SPARC controls intracellular signaling pathways in neurons to impact AMPAR trafficking remains to be determined.

### Chondroitin-sulfate proteoglycans (CSPGs)

Astrocytes contribute to the ECM surrounding neurons by secreting proteoglycans such as CSPG. Frischknecht et al. (2009) showed that digestion of the ECM backbone with hyaluronidase results in increased AMPAR mobility in the membrane and increased short-term plasticity (STP), suggesting that the ECM restricts exchange of desensitized synaptic AMPARs with naïve extrasynaptic AMPARs during STP (Frischknecht et al., 2009). Since CSPG levels increase during postnatal development, they may contribute to synapse maturation by limiting plasticity (Pizzorusso et al., 2002). Consistent with this, digestion of ECM increased synapse number in a hippocampal/astrocyte co-culture system and electrophysiological recordings indicated that the synapses were immature (Pyka et al., 2011). Thus, components of the ECM like CSPG and its interacting proteins are required to stabilize synaptic AMPARs and promote synapse maturation. It will be interesting to determine how CSPG expression or secretion from astrocytes is regulated to control synaptic AMPARs and maturation.

### Glypicans (Gpcs)

Glypicans are conserved heparan sulfate proteoglycans (HSPGs) comprising 6 family members in mammals that are anchored to the plasma membrane via glycosylphosphatidylinositol links. Allen et al. (2012) biochemically isolated Gpc4 and Gpc6 from astrocyte-conditioned media as factors that induce functional retinal ganglion cell (RGC) synapses. Both Gpc4 and Gpc6 promote cell surface clustering of GluA1-containing AMPARs and increased excitatory transmission (Allen et al., 2012). Knock-out of Gpc4 in mice decreases GluA1 and reduces excitatory transmission in the developing hippocampus. The authors proposed that glypicans induce immature synapses containing GluA1 which then subsequently mature by incorporating GluA2/3 and GluA4 subunit-containing AMPARs. Although glypicans can be proteolytically cleaved and released from the plasma membrane, it is not clear how astrocytes regulate the release of glypicans.

Other studies have revealed the mechanism by which Gpc4 regulates AMPARs. Mammalian glypicans and the *Drosophila* glypican homolog Dally like interacts with Leukocyte common antigen-related (LAR) receptor tyrosine phosphatases (RPTPs) family members (Johnson et al., 2006; Takahashi and Craig, 2013; Ko et al., 2015) and postsynaptic Leucine-Rich Repeat TM4 (LRRTM4) (de Wit et al., 2013; Siddiqui et al., 2013). These interactors promote excitatory synapse formation and function in mice (Dunah et al., 2005; Linhoff et al., 2009; Siddiqui et al., 2013; Soler-Llavina et al., 2013) by increasing surface GluA1-containing AMPARs (Schwenk et al., 2012; Shanks et al., 2012; Siddiqui et al., 2013). Additionally, astrocyte-secreted Gpc4 stimulates release of NPTX1 to promote AMPAR clustering at synapses (see above). Disruption of the NPTX1-AMPAR interaction with antibodies blocks Gpc4-mediated synapse formation, and loss of Gpc4 or LAR family member RPTPδ results in accumulation of NPTX1 in presynaptic terminals, reduced GluA1 clusters and decreased synapse number (Farhy-Tselnicker et al., 2017). These data suggest a model whereby astrocyte-released Gpc4 interacts with presynaptic RPTPδ resulting in secretion of NPTX1 from neurons and postsynaptic AMPAR clustering.

### Chordin-like-1 (Chrdl1)

Excitatory synapse maturation is marked by a switch from calcium-permeable GluA1-containing to calcium-impermeable GluA2-containing AMPARs. Blanco-Suarez et al. (2018) identified Chordin-like 1 (Chrdl1) as an astrocyte-secreted factor that promotes synapse maturation by increasing surface levels of GluA2-containing AMPARs at synapses on rat RGCs, thus inducing the switch from calcium-permeable to calcium-impermeable AMPARs (Blanco-Suarez et al., 2018). Interestingly, Chrdl1 knock-out mice exhibit a shift toward immature synapses which is associated with increased plasticity in the visual cortex. The authors propose a model where Gpcs act first to promote clustering of GluA1-containing AMPARs at immature synapses followed by Chrdl-1 which promotes the switch to GluA2-containing AMPARs typically associated with mature synapses. Consistent with this model, astrocyte expression of Gpc4 and Gpc6 is highest in the first two postnatal weeks when immature synapses are developing while Chrdl1 peaks in the cortex later at the time of synapse maturation in mice (Cahoy et al., 2008; Allen et al., 2012). It remains to be determined how Chrdl1 secretion is regulated and how Chrdl1 regulates intracellular trafficking to promote GluA2-containing AMPARs. Given that CSPGs also promote synapse maturation, it will be informative to investigate how Chrdl-1 and CSPG interact to regulate maturation.

### Tumor necrosis factor α (TNF-α)

The proinflammatory cytokine TNFα is secreted from astrocytes or microglia and promotes surface levels of AMPARs in hippocampal neurons *in vitro* and *in vivo* (Beattie et al., 2002). TNFα regulation of AMPARs is not involved in hippocampal LTP or LTD, but is required for homeostatic synaptic scaling (Stellwagen and Malenka, 2006) in which chronic changes in synaptic activity result in a global adjustment of synaptic strength by altering AMPAR levels (O’Brien et al., 1998; Turrigiano et al., 1998). Synaptic scaling up is blocked if TNFα is scavenged by soluble TNFR1 or if neurons are co-cultured with TNFα knock out glia (Stellwagen and Malenka, 2006). Mechanistically, TNFα increases the exocytosis of GluA2-lacking AMPARs in hippocampal and cortical neurons (Ogoshi et al., 2005; Stellwagen and Malenka, 2006; He et al., 2012) via PI3 kinase (Stellwagen and Malenka, 2006) while simultaneously promoting the internalization of GABA receptors (Stellwagen et al., 2005). The effects of TNFα are brain region-specific. TNFα has the opposite effect on AMPARs in the mouse dorsolateral striatum where it promotes internalization of GluA1-containing AMPARs from medium spiny neurons and reduces corticostriatal synaptic strength (Lewitus et al., 2014). In the striatum, TNFα treatment activates protein phosphatase 1 (PP1) via DARPP-32 which promotes AMPAR endocytosis by increasing the dephosphorylation of GluA1 on S845 and S831.

### Wingless (Wg)/Wnt

Wingless (Wg)/Wnt is secreted from neurons and glia and regulates pre- and postsynaptic development via the receptor *Frizzled* at the glutamatergic *Drosophila* neuromuscular junction (NMJ) (Packard et al., 2002; Mathew et al., 2005; Miech et al., 2008). Loss of Wg results in decreased synapse numbers and abnormal glutamate receptor (GluR) distribution (Packard et al., 2002). Interestingly, Wg is secreted from glia in addition to motor neurons at the *Drosophila* NMJ. Loss of Wg in neurons or subperineurial glia results in increased GluR cluster size and intensity, however, these synapses are correlated with reduced quantal content. Thus, Wg secreted from both glia and neurons is required for the normal distribution and function of GluRs. Interestingly, the source of Wg matters because Wg secreted from motor neurons, but not glia, regulates NMJ size, suggesting that neuron-derived Wg regulates NMJ structure. Although it is not clear how the Wg receptor Frizzled signals to regulate GluR trafficking, vertebrate Wnt7a promotes synapse formation in hippocampal neurons via postsynaptic CaMKII (Ciani et al., 2011) which is known to promote synaptic AMPAR levels during plasticity (Hayashi et al., 2000).

## Factors secreted from other tissues

### Vascular endothelial growth factor (VEGF)

A recent study in *C. elegans* showed that the VEGF homolog PVF-1 is secreted from muscle and regulates surface levels of the calcium-permeable AMPAR GLR-1 in upstream neurons (Luth et al., 2021). Interestingly, PVF-1/VEGF is expressed and released from muscle to mediate GLR-1 surface levels in pre-motor interneurons that reside two synaptic layers upstream of the neuromuscular junction. Loss of the VEGF Receptor (VEGFR) homologs VER-1 and VER-4, or PVF-1 reduces GLR-1 cell surface levels, resulting in impaired glutamate-dependent locomotor behaviors (Luth et al., 2021). Local recycling of GLR-1 to the plasma membrane is notably unchanged in VER mutants, consistent with the idea that VEGFR signaling in *C. elegans* promotes long-range AMPA receptor trafficking, perhaps via retromer (Luth et al., 2021).

In vertebrates, VEGF is expressed in vascular endothelial cells throughout the body including in the brain and spinal cord (Licht et al., 2011), but also in neurons (Ogunshola et al., 2002; Schiera et al., 2007), microglia (Bartholdi et al., 1997) and astrocytes (Ijichi et al., 1995). VEGF is also expressed in multiple muscle cell-types such as cardiac myocytes (Levy et al., 1995), vascular smooth muscle (Ishida et al., 2001) and skeletal muscle (Germani et al., 2003). VEGF expression can be induced by ischemia and, in the case of skeletal muscle, is known to both promote angiogenesis and act in an autocrine manner to regulate myoblast function to promote new muscle growth (Germani et al., 2003). Interestingly, VEGFR family members are expressed in neurons and glia and can be induced in astrocytes and microglia following injury (Ogunshola et al., 2002; Choi et al., 2007a,b).

Several studies indicate that VEGF regulates glutamatergic transmission in vertebrates (Cammalleri et al., 2011; Licht et al., 2011; De Rossi et al., 2016). Inhibiting or stimulating VEGF signaling in adult mice has respective effects on LTP in the hippocampus and glutamate-dependent fear conditioning (Licht et al., 2011). VEGFRs localize to the postsynaptic density of mouse hippocampal neurons, and although VEGF alone failed to increase AMPA receptor trafficking or LTP (Cammalleri et al., 2011; De Rossi et al., 2016), co-administration of VEGF and NMDA activated PKC and CaMKII, increased the delivery of calcium-permeable GluA1-containing AMPARs to synapses, and increased excitatory synapse number. Conversely, conditional knockout of VEGFR2 in neurons impaired LTP and fear-related memory consolidation, without altering basal transmission (De Rossi et al., 2016). Collectively, these findings suggest that in the hippocampus, VEGFR2 signaling may promote activity-dependent strengthening of glutamatergic transmission and resultant behavior by promoting the synaptic incorporation of AMPARs. Together these studies indicate that VEGF regulates AMPAR levels and trafficking in vertebrates and invertebrates, however, the source of VEGF in vertebrates is not clear. Given that PVF-1/VEGF is secreted from muscle to regulate AMPARs in *C. elegans*, it is tempting to speculate that muscle-secreted VEGF may, under some circumstances, also act at a distance to regulate AMPARs in the brain in vertebrates.

### Fibronectin type III domain-containing protein 5 (FNDC5)/Irisin

Muscle activity during exercise has beneficial effects on energy metabolism and brain function (Cotman et al., 2007; Mattson, 2012). Interestingly, previous studies identified irisin as a muscle-released factor that signals to the brain. FNDC5 is a transmembrane protein expressed in the brain and skeletal muscle that is cleaved and released as a peptide called irisin during exercise. Irisin enters the circulation and directs fat metabolism and thermogenesis and also mediates many of the beneficial effects of exercise on cognition (Boström et al., 2012). Peripheral injection of FNDC5 or irisin increases irisin protein levels in the hippocampus without altering irisin mRNA levels in the brain (Wrann et al., 2013; Lourenco et al., 2019; Islam et al., 2021), suggesting that irisin can cross the blood-brain barrier. Once in the brain, irisin induces the expression of BDNF in the hippocampus (Wrann et al., 2013), which as discussed above, can regulate AMPAR expression and trafficking. Interestingly, BDNF expression is increased after exercise in the hippocampus (Vaynman et al., 2004; El Hayek et al., 2019; Lourenco et al., 2019) and also in skeletal muscle (Cuppini et al., 2007; Szuhany et al., 2015) and can be released into the circulatory system (Pan et al., 1998; Matthews et al., 2009).

## Discussion

This mini-review highlights secreted protein factors that regulate trafficking or clustering of AMPAR subunits and discusses their effects on glutamatergic transmission and plasticity (Table 1). Several factors are secreted in response to activity either from neurons or glia and are thus ideal for coordinating synapse development or plasticity in an activity-dependent manner. Regulation of the expression and release of secreted factors potentially provides a multitude of spatio-temporal mechanisms to control neuronal development and function. Depending on their range of action, secreted factors could act locally to coordinate subsets of synapses within a neuron or more globally to coordinate groups of glia or neurons in a circuit or region of the brain. Longer range factors may act more distally in another region of the brain and still signal to a restricted subset of neurons that express their specific cognate receptor, enabling precise long-distance signaling. However, since most studies described in this review typically focus on one neuronal cell type or one region of the brain, their full complement of target cells within the brain are not known.

We also describe a role for PVF-1/VEGF as a muscle-secreted factor in *C. elegans* that regulates AMPAR levels in a distal neuron that coordinates locomotion. Although VEGF is also expressed and released from muscle in vertebrates, it’s role in signaling to the brain has not been investigated. However, the fact that muscle cells release hundreds of factors, many of which are stimulated by contraction that may mediate the beneficial effects of exercise on metabolism and brain function (Görgens et al., 2015) and that studies in *Drosophila* and mammals have revealed hundreds of secreted proteins that may mediate inter-organ communication including between peripheral tissues and the brain (Droujinine and Perrimon, 2016; Severinsen and Pedersen, 2020), raises the intriguing possibility that there may be other factors secreted from peripheral tissues that regulate glutamatergic synapses in the brain.

Many open questions remain. Although neuronal activity can regulate the release of some of these factors, we do not understand how they are packaged into vesicles, the mechanisms that regulate their secretion, or how their range of action might be modulated. Similarly, although we have some idea how a few of these factors regulate AMPAR trafficking, for the vast majority, the molecular mechanisms by which they impinge on intracellular trafficking pathways (lateral diffusion, clustering, insertion, removal, degradation, transport, etc.) to control synaptic AMPAR levels are unknown. It will also be interesting to determine when these factors are expressed and released either during development or in the mature brain and if they are expressed in a region-specific manner. Finally, although this review largely focused on the secretion of soluble factors, there are many membrane-associated or transmembrane proteins, like FNDC5 and glypicans that undergo cleavage to release extracellular domains (Tien et al., 2017) that may have signaling or regulatory functions that remain to be discovered. It will not be surprising if more extracellular factors that regulate AMPAR trafficking and glutamatergic synapses will be discovered that are secreted from both neuronal and non-neuronal cell types in the brain, and perhaps also from peripheral tissues.

## Author contributions

BR: Writing – original draft, Writing – review and editing. EL: Writing – original draft, Writing – review and editing. SM: Writing – original draft, Writing – review and editing. PJ: Conceptualization, Writing – original draft, Writing – review and editing.
